# Accuracy of Optical Heart Rate Sensing Technology in Wearable Fitness Trackers for Young and Older Adults: Validation and Comparison Study

**DOI:** 10.2196/14707

**Published:** 2020-04-28

**Authors:** Hsueh-Wen Chow, Chao-Ching Yang

**Affiliations:** 1 Graduate Institute of Physical Education, Health & Leisure Studies National Cheng Kung University Tainan City Taiwan

**Keywords:** pulse, photoplethysmography, wearable device, aerobic exercise

## Abstract

**Background:**

Wearable fitness trackers are devices that can record and enhance physical activity among users. Recently, photoplethysmography (PPG) devices that use optical heart rate sensors to detect heart rate in real time have become popular and help in monitoring and controlling exercise intensity. Although the benefits of using optical heart rate monitors have been highlighted through studies, the accuracy of the readouts these commercial devices generate has not been widely assessed for different age groups, especially for the East Asian population with Fitzpatrick skin type III or IV.

**Objective:**

This study aimed to examine the accuracy of 2 wearable fitness trackers with PPG to monitor heart rate in real time during moderate exercise in young and older adults.

**Methods:**

A total of 20 young adults and 20 older adults were recruited for this study. All participants were asked to undergo a series of sedentary and moderate physical activities using indoor aerobic exercise equipment. In this study, the Polar H7 chest-strapped heart rate monitor was used as the criterion measure in 2 fitness trackers, namely Xiaomi Mi Band 2 and Garmin Vivosmart HR+. The real-time, second-by-second heart rate data obtained from both devices were recorded using the broadcast heart rate mode. To critically analyze the results, multiple statistical parameters including the mean absolute percentage error (MAPE), Lin concordance correlation coefficient (CCC), intraclass correlation coefficient, the Pearson product moment correlation coefficient, and the Bland-Altman coefficient were determined to examine the performances of the devices.

**Results:**

Both test devices exhibited acceptable overall accuracy as heart rate sensors based on several statistical tests. Notably, the MAPE values were below 10% (the designated threshold) in both devices (Garmin_Young_=3.77%; Garmin_Senior_=4.73%; Xiaomi_Young_=7.69%; and Xiaomi_Senior_=6.04%). The scores for reliability test of CCC for Garmin were 0.92 (Young) and 0.80 (Senior), whereas those for Xiaomi were 0.76 (Young) and 0.73 (Senior). However, the results obtained using the Bland-Altman analysis indicated that both test optical devices underestimated the average heart rate. More importantly, the study documented some unexpected outlier readings reported by these devices when used on certain participants.

**Conclusions:**

The study reveals that commonly used optical heart rate sensors, such as the ones used herein, generally produce accurate heart rate readings irrespective of the age of the user. However, users should avoid relying entirely on these readings to indicate exercise intensities, as these devices have a tendency to produce erroneous, extreme readings, which might misinterpret the real-time exercise intensity. Future studies should therefore emphasize the occurrence rate of such errors, as this will likely benefit the development of improved models of heart rate sensors.

## Introduction

### Growing Popularity and Functions of Wearable Fitness Trackers

Wearable fitness trackers have gained popularity worldwide, and their annual sales continue to grow [1,2]. These trackers were listed as the No. 1 fitness trends in the years 2016, 2017, 2019, and 2020 in a worldwide survey conducted by the American College of Sports and Medicine [3-6]. The advantages of these wearable devices are that they are convenient to use and measure various parameters noninvasively. In addition, they allow the users to monitor their daily physical activities in a free-living environment instead of controlled laboratory settings.

Earlier versions of fitness trackers, equipped with triaxial accelerometers and a gyroscope, could sense motions made by the users, monitor their activity metrics, and provide estimated information such as walking and running in terms of steps or distance, energy expenditure, sedentary time, sleep patterns, and activity routes (with GPS function). Most of these fitness trackers were placed on the wrist. The users obtained the real-time information from the display on the trackers or received feedback through connected mobile phone apps.

The recent application of photoplethysmography (PPG) in wrist-based wearable fitness trackers has enabled newer versions of fitness trackers to detect heart rates. This breakthrough provides several benefits. First, heart rate is a vital component in cardiovascular fitness assessments and an important parameter in exercise training programs [7]. Second, resting heart rate is also a widely used parameter for general health assessments to detect cardiovascular diseases [8]. Thus, the development of fitness trackers that have heart rate detection technologies has brought about several additional benefits that were absent in older models.

PPG measures heart rates based on the changes in vascular blood flow during the cardiac cycle [9]. It has previously been applied in medical devices such as oximeters [10]. This technology has since been integrated and commercialized as optical heart rate monitors by companies such as Mio and Omron. The number of commercial companies producing such devices has gradually grown in the last 5 years (ie, Apple Watch, Fitbit, and Garmin), along with the design and development of such products and research [1,2,10-16].

### Validation of Fitness Trackers

Despite the growing popularity and functions of these fitness trackers and substantial investments in commercial advertisements, many users have expressed concerns regarding the data accuracy of these trackers [17]. Inaccurate and inconsistent readings are major reasons for negative user experiences, which discourage the continued use of these devices [17-20]. The concerns regarding the data accuracy of these trackers influence the users in terms of their perceptions of personal health and program interventions or research evaluations that adopt these devices.

Most commercially available fitness trackers use step counts as a parameter to indicate the level of physical activity. The step-count function of these devices has been widely scrutinized in studies examining their accuracy [21-23]. Importantly, while generally producing accurate results, these devices did not report reliable step-count readings in certain conditions, such as slow walking or while performing unnatural hand movements [21-24]. A systematic review investigated the validity and reliability of Fitbit and Jawbone trackers. The results revealed that most studies validated the tracker accuracy and indicated that it had a higher accuracy for step counts, followed by that for distance and physical activity and finally for energy consumption and sleep [23]. Nevertheless, most studies recommend caution when deriving energy expenditure estimations directly using these readings [11,13,25,26]. In addition, studies have started to examine the validity and reliability of the fitness trackers among older adults instead of young adults because they might present different movements such as gait patterns or speeds [27,28].

### Accuracy of Optical Heart Rate Monitoring

The accuracy of heart rate displayed on the fitness trackers with optical heart rate monitors has also been investigated [11,14,16,29-31]. Common research methods for the development of these optical heart rate monitors involve fitness assessments using basic indoor training equipment such as treadmills, stationary cycles, and sometimes elliptical machines. This type of study allows researchers to evaluate the feasibility of implementing optical heart rate monitors in aerobic training for the general population [1,2,10-16].

Previous studies have reported that, generally, optical sensing fitness trackers have acceptable accuracy. However, the accuracy might vary across brands [16,31] in terms of activity patterns or speed, exercise intensities [10,14,31], skin tone [10], room temperature [32], placement of sensors [29], or compression-induced and motion-induced artifacts [13,32-34]. For example, in a study conducted by Boudreaux et al [13], participants wore 8 different fitness trackers, and an increase in exercise intensity reduced the accuracy of heart rate measurement. In another validation study, the measured heart rate showed a minor deviation compared with the actual heart rate in participants with a dark skin tone [30].

Although the adoption of heart rate fitness trackers with optical heart rate sensors in the medical field is still debatable [12,35], there have been several lawsuits regarding the accuracy of heart rate information [36,37]. Assessing the reliability and validity of the heart rate readings provided by these trackers is essential because they are vital in clinical settings, and these trackers have been increasingly accepted by consumers as a tool for self-monitoring or in many intervention programs for health management [11,14].

### Research Gaps

Owing to the limitations on raw data acquisition in commercial fitness trackers, previous studies have only used average heart rate data [14] or manually recorded the heart rate at certain intervals [11]. However, averaging the heart rate or recording it at a certain time point is problematic because both fail to represent any change or variability [38]. Studies that have compared continuous heart rate in more detail revealed that evaluating the accuracy of these test devices at a second-by-second level is difficult [2]. One study used video recording to manually determine the second-by-second heart rate, which was a labor-intensive and time-consuming method [12]. Moreover, potential variables such as age, ethnicity, and gender were not considered in earlier studies [2,14]. For example, a majority of the participants of several studies that have been conducted in the US-European regions were white (Fitzpatrick skin type I or II) [2,12,16]. PPG technology uses an optical sensor that illuminates light and measures the change in light absorption by the skin, which varies with change in blood volume; thus, the accuracy of heart rate monitoring using PPG is subject to skin structures [39]. Typically, the skin changes with age, that is, “fine wrinkles, roughness, mottled hyperpigmentation, dilated blood vessels, and loss of skin tone” are observed [40]. In addition, age-related changes such as arterial stiffness can influence the pulse shape in PPG [32]. Therefore, appropriate validation of these devices for different age groups among non-white participants is imperative.

### Aim of the Study

This study evaluated the heart rate reading performances of 2 commercially available fitness trackers in various settings using a second-by-second data acquisition approach. Moreover, to determine whether age would generate discrepancies in the readouts, young and senior participants were characterized separately. This study was conducted in Taiwan to validate 2 trackers used by the yellow skin tone population (Fitzpatrick skin type III or IV) [41,42].

## Methods

### Participants

To determine a credible sample size for achieving statistical power in the intraclass coefficient correlation (ICC) test, this study used R package (ICC.Sample. Size, GPL-3; 2015, R core team, R Foundation for Statistical Computing). Based on the formula proposed by Zou [43], the number of participants (n) required for achieving a target power of 0.90 was 8. Therefore, this study involved 20 adults aged 65 years and above (Senior) and 20 adults aged between 20 years and 26 years (Young). All participants had no clinical history of cardiovascular diseases, neurological disorders, lower limb injuries, or any other factors that would render them unfit to perform the exercise. To ensure consistency, individuals with tattoos or birthmarks on the position where the device was to be worn were not included in the study. To minimize possible sex-driven discrepancies, the sex ratio in both the Senior and Young groups was kept identical (20:20).

### Research Device

This study used the Polar heart rate strap (H7, Polar Electro Oy), widely used as the criterion for measuring heart rate in sports science studies [2,44]. The optical fitness trackers selected for this study were Xiaomi Mi Band 2 (Xiaomi Cooperation) and Garmin Vivosmart HR+ (Garmin International Inc) because these 2 fitness trackers share a significant market share in the Asia Pacific region, which is expected to grow. Mi Band 2 was equipped with a PPG module (with 2 LED lights) and an accelerometer to detect heart rate and sense motion. Vivosmart HR+ was also equipped with a PPG module (with 3 LED lights) and an accelerometer. In addition, GPS chips are embedded in the Vivosmart HR+ for measuring the travel distance during outdoor exercises.

Both the devices provided information regarding step counts, energy expenditure, notification for breaking up the prolonged sedentary time, and smart notifications, and both claimed accurate heart rate detection. In addition, the 2 devices had the broadcast heart rate mode, a feature that enables the transmission of second-by-second heart rate data through Bluetooth or ANT+ to the paired receiving device, and served a similar function of the conventional heart rate strap. Moreover, wrist-based fitness trackers were easy to wear and remove and, thus, eased the discomfort of wearing chest straps for monitoring the real-time heart rate during traditional exercise and fitness training programs or interventions [10,45]. Specifically, PPG fitness trackers provide pulse rate data that are obtained with an increase or decrease in blood pressure in the arteries because of the contraction and relaxation of the heart, thus leading to a noticeable pulse. Although the signals of pulse waveforms are different from those of heartbeat waveforms, the pulse rate can be analyzed to represent the heart rate [32]. The term heart rate has been used in this study in line with many studies on heart rate fitness trackers [2,10-12,15,29,30,38,46]. Hence, in this study, the heart rate will be used in its broadest sense to refer to the readings from the optical fitness trackers.

**Figure 1:**
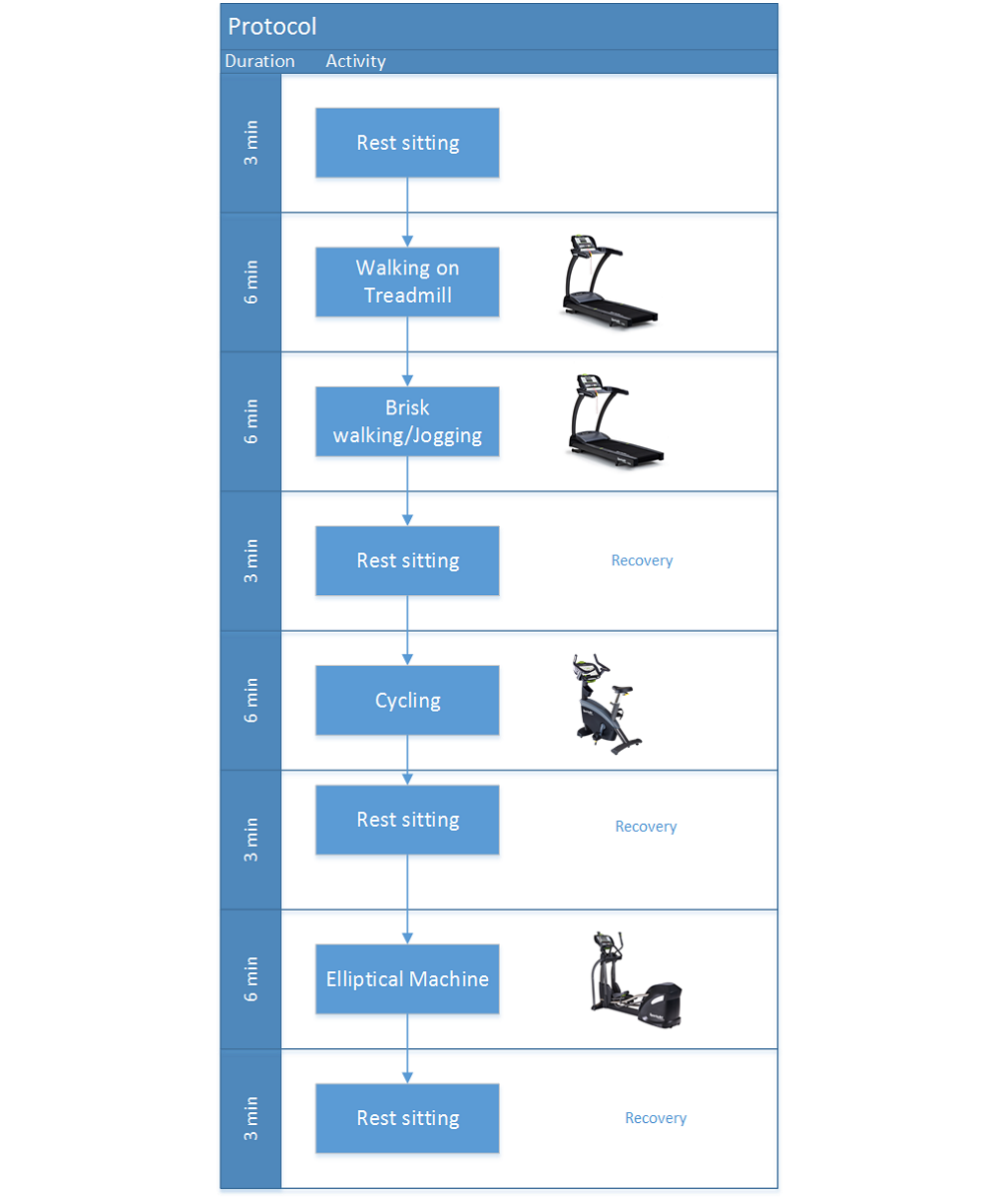
Exercise protocol.

The second-by-second heart rate data-receiving app Cardio Training (Angelfmarcos) used in this study was acquired from the Android platform. The equipment adopted in this study included 3 indoor aerobic fitness equipment: treadmill, upright stationary bike, and elliptical machine (Figure 1). These types of equipment were widely demonstrated in the previous exercise protocols and proved to be ideal and safe for aerobic training [2,10,47-49].

### Procedure

#### Before the Trial

The study was approved by the Institutional Review Board of the National Cheng Kung University Hospital (IRB number: B-ER-106-134). All participants gave written consent to participate in the trial and were provided a detailed explanation of the complete research protocol before the commencement of the study. All participants were given the option to voluntarily withdraw from the trial at any time during the study.

Polar H7 chest-strapped heart rate monitors and wrist-strapped optical fitness trackers were fixed onto the participants by the researcher according to the manufacturer instructions. Next, the broadcast heart rate mode of the optical fitness trackers was activated by the researcher simultaneously. Data transmission to the tablets or mobile phones was then checked.

#### Exercise Protocol

Initially, participants were asked to be seated quietly for 15 min to record their resting heart rates (HR_rest_) using the Polar H7 heart rate monitors. The general formula (220−age in years) was used for calculating the maximal heart rate (HR_max_) of each individual. Based on the HR_rest_ and HR_max_, a personalized *moderate exercise intensity* was determined for each participant. This was defined by 40% to 60% of heart rate reserve, which is the difference between HR_max_ and HR_rest_ [50]. Finally, participants were led to the exercise area and shown the proper usage and adjustment of the specific fitness equipment.

To evaluate the heart rate detection accuracy of the test devices during different activities, participants were instructed to perform a sequence of sedentary and aerobic exercises [2,10]. The sequence was divided into phases, and heart rates were recorded using the Cardio Training app at each phase. The participants were initially guided to adjust the workout level of equipment accordingly to prevent exhaustion before the end of the trial. Specifically, the measurement began with the participants seated (rest sitting), which represented a typical sedentary behavior. Next, participants were asked to walk on the treadmill for 6 min (the warm-up phase) before engaging in more vigorous exercises. Every period of the exercise phase lasted for 6 min. The step-by-step protocol is presented in Figure 1. Rest sitting time was given to the participants between each phase, during which the heart rate measurement would continue.

During the exercise phases, participants were encouraged to maintain moderate exercise intensity. Real-time feedback and instructions were given by the researcher verbally as guided by the heart rate data acquired from the Polar H7 heart rate monitor. Except in circumstances where the participant deviated from moderate exercise intensity, in which the resistance level was adjusted accordingly, no further intervention by the researcher was made during the entire trial.

#### Statistical Analyses

Using the Cardio Training app, the second-by-second heart rate data generated from the trials were exported as CSV files. A total of 2161 readings, corresponding to 2161 seconds (including the first reading at the beginning of the protocol), were obtained and recorded for each participant. Compared with previous studies, in which heart rate measurements were less frequent (ie, every 15 seconds/every minute or only at the end of each exercise phase) [11,15,16], the statistical results produced from the current dataset are likely to be more representative because they enabled the researchers to discern some potential outlier readings. To compare the accuracy of test devices, various statistical methods were chosen based on recommendations from relevant studies [2,10,26,38,51]. All statistical tests were performed using SPSS 18.0 (IBM) and MedCalc statistical software (MedCalc).

#### Reliability

To compare the reliability between the criterion measurement device (Polar H7) and the 2 test optical fitness trackers, 3 reliability tests were used, namely the Lin concordance correlation coefficient (CCC), Pearson product moment correlation coefficient (PPMCC), and ICC tests (two-way mixed, single measures, and absolute agreement). Discrepant standards were used for interpreting the results of the reliability correlation tests. For instance, Gillinov et al [2] set the CCC value greater than 0.80 to represent acceptable reliability, whereas Boudreaux et al [13] set ICC values from 0.60 to 0.75 to represent moderate reliability and from 0.75 to 0.90 to indicate superior reliability. Moreover, other studies on applied sports science have proposed a slightly different version of interpreting ICC values: values between 0.50 and 0.75 indicated moderate reliability, whereas other thresholds were the same [52]. This study used all 3 of the aforementioned reliability tests.

#### Analysis of Paired Difference

Paired absolute differences from mean absolute error (MAE) and mean absolute percentage error (MAPE) were determined to reveal the differences between the criterion measurement and measurements generated by the test devices among respective age groups and during different phases of the exercise (MAPE is calculated by subtracting the HR readings from the Mi or Garmin from the Polar H7 and then dividing by the Polar H7). Results with error values below 10% were considered reliable [13].

#### Bland-Altman Analysis

To determine the agreement of the criterion measurement and measurements generated by the optical fitness trackers, Bland-Altman analysis was applied to explore the mean bias and 95% CI limits of agreement. The results from different age groups and during different phases of the exercise were analyzed and represented graphically.

## Results

### Reliability of Examined Devices

The results of MAE, MAPE, and correlation tests from both the Young and Senior groups are shown in Tables 1 and 2. In the Young group, the Garmin device achieved MAPE values of less than 10% in all the conditions tested (Table 1), indicating that overall, the heart rate readings produced by the Garmin device were reliable [2,13]. By contrast, whereas the Xiaomi device generally achieved MAPE values of less than 10%, it did not do so during cycling and elliptical phases (Table 1), suggesting that the reliability of the Xiaomi device was likely influenced by the types of activities performed.

In the Senior group, the performances of both test devices during different activities were reliable (MAPE values below 10%, Table 1). Notably, the MAPE values achieved by the Xiaomi device were, on average, higher than those produced by the Garmin device, indicating that the Xiaomi product was overall less reliable than the Garmin one. However, the standard deviation of MAPE achieved by the Garmin device was higher in the Senior group (SD_Senior_=10.49%) than in the Young group (SD_Young_=6.9%; Table 1), suggesting that the reliability of the Garmin device was likely affected by age differences and that it became less reliable in the older population.

**Table 1:** Mean absolute percentage error (MAPE) and Bland-Altman analyses of heart rate readings of the Young and Senior groups during different activity phases.

Group, activity, number of readings, and device				MAPE analysis, mean (SD)		Bland-Altman analysis
				Mean absolute error (bpm)	Mean absolute percentage error	Mean difference (lower to upper limits of agreement)
**Young**						
	**Rest**					
		**3620**				
			Ga^a^	2.98 (3.14)	3.96 (4.17)	−1.4 (−9.4 to 6.6)
			Mi^b^	3.27 (4.48)	4.46 (6.05)	0 (−10.9 to 10.8)
	**Walking**					
		**7200**				
			Ga	3.35 (4.73)	3.77 (5.29)	0.2 (−11.5 to 11.2)
			Mi	6.39 (7.93)	7.46 (9.93)	3.7 (−14.8 to 22.3)
	**Running**					
		**7200**				
			Ga	3.48 (7.66)	2.85 (6.29)	−2.6 (−18.3 to 13.1)
			Mi	10.41 (12.99)	8.32 (10.54)	6.7 (−23.1 to 36.6)
	**Cycling**					
		**7200**				
			Ga	6.19 (14.41)	4.92 (10.79)	−5.7 (−34.3 to 23.0)
			Mi	14.05 (20.56)	10.93 (15.36)	−13.4 (−54.5 to 27.8)
	**Elliptical**					
		**7200**				
			Ga	3.06 (5.11)	2.52 (4.32)	−2.0 (−13.0 to 9.0)
			Mi	14.06 (19.73)	10.77 (14.88)	−13.3 (−53.0 to 26.4)
	**Recovery**					
		**10,800**				
			Ga	4.40 (7.22)	4.38 (6.85)	1.0 (−15.4 to 17.5)
			Mi	4.86 (7.90)	4.73 (7.60)	0.5 (−17.7 to 18.7)
	**Total**					
		**43,220**				
			Ga	4.03 (8.21)	3.77 (6.90)	−1.6 (−19.3 to 16.1)
			Mi	8.85 (14.46)	7.69 (11.66)	−2.6 (−35.5 to 30.3)
**Senior**						
	**Rest**					
		**3620**				
			Ga	1.96 (3.53)	2.45 (4.11)	−1.0 (−8.7 to 6.6)
			Mi	4.03 (6.54)	5.59 (9.67)	1.2 (−13.7 to 16.1)
	**Walking**					
		**7200**				
			Ga	6.72 (10.56)	7.06 (10.96)	4.3 (−18.8 to 27.3)
			Mi	8.09 (12.77)	8.69 (13.85)	2.8 (−26.3 to 31.9)
	**Running**					
		**7200**				
			Ga	2.7 (4.36)	2.54 (4.08)	−1.4 (−11.0 to 8.3)
			Mi	7.46 (16.73)	7.02 (16.14)	3.6 (−31.6 to 38.8)
	**Cycling**					
		**7200**				
			Ga	3.85 (11)	3.65 (9.69)	−3.2 (−25.2 to 18.7)
			Mi	3.91 (7.5)	3.78 (6.92)	−2.4 (−18.3 to 13.6)
	**Elliptical**					
		**7200**				
			Ga	5.19 (10.94)	5.04 (11.51)	0.6 (−23.1 to 24.3)
			Mi	7.31 (11.55)	6.38 (9.36)	−5.2 (−30.0 to 19.6)
	**Recovery**					
		**10,800**				
			Ga	5.43 (11.58)	5.92 (13.48)	2.2 (−22.4 to 26.9)
			Mi	4.85 (8.46)	5.05 (8.97)	0.1 (−19.0 to 19.3)
	**Total**					
		**43,220**				
			Ga	4.6 (9.93)	4.73 (10.49)	0.5 (−20.9 to 21.9)
			Mi	6.02 (11.39)	6.04 (11.33)	−0.1 (−25.3 to 25.2)

^a^Ga: Garmin Vivosmart HR^+^.

^b^Mi: Xiaomi Mi Band 2.

The data revealed that the Garmin device achieved CCC values above the designated threshold (0.80) in both age groups (Table 2), suggesting that it was generally accurate. By contrast, the Xiaomi device failed to achieve overall CCC values above the designated threshold in both age groups (Table 2), indicating that it exhibited suboptimal accuracy in heart rate sensing. Notably, similar to the MAPE values described earlier, whereas the Xiaomi device achieved identical CCC values in both age groups (CCC_Young_=0.73; CCC_Senior_=0.73), the Garmin device’s CCC values fluctuated between the 2 age groups (CCC_Young_=0.93; CCC_Senior_=0.80; Table 2), indicating that its accuracy was also likely influenced by age differences. Taken together, these data suggest that the Garmin device, in general, produced more reliable and accurate heart rate readings than the Xiaomi one.

**Table 2:** Correlation analyses of heart rate readings of Young and Senior groups during different activity phases.

Group, activity, number of readings, and device				Correlation		
				CCC^a^	ICC^b^	PPMCC^c^
**Young**						
	**Rest**					
		**3620**				
			Ga^d^	0.9037	0.9038	0.914
			Mi^e^	0.8475	0.8475	0.8475
	**Walking**					
		**7200**				
			Ga	0.8577	0.8577	0.8598
			Mi	0.6074	0.6074	0.6461
	**Running**					
		**7200**				
			Ga	0.8552	0.8552	0.8858
			Mi	0.5428	0.5428	0.6185
	**Cycling**					
		**7200**				
			Ga	0.5888	0.5889	0.6569
			Mi	0.2874	0.2874	0.4037
	**Elliptical**					
		**7200**				
			Ga	0.9104	0.9104	0.9261
			Mi	0.3267	0.3267	0.4734
	**Recovery**					
		**10,800**				
			Ga	0.8972	0.8972	0.8993
			Mi	0.8863	0.8863	0.8931
	**Total**					
		**43,220**				
			Ga	0.9254	0.9254	0.9277
			Mi	0.7603	0.7603	0.767
**Senior**						
	**Rest**					
		**3620**				
			Ga	0.9262	0.9262	0.9306
			Mi	0.7320	0.7321	0.7369
	**Walking**					
		**7200**				
			Ga	0.5925	0.5925	0.722
			Mi	0.4464	0.4464	0.5469
	**Running**					
		**7200**				
			Ga	0.9246	0.9246	0.9311
			Mi	0.4592	0.4593	0.5288
	**Cycling**					
		**7200**				
			Ga	0.4799	0.4799	0.5129
			Mi	0.6856	0.6856	0.7081
	**Elliptical**					
		**7200**				
			Ga	0.7516	0.7516	0.7684
			Mi	0.6612	0.6612	0.6992
	**Recovery**					
		**10,800**				
			Ga	0.7055	0.7055	0.7253
			Mi	0.7929	0.793	0.7934
	**Total**					
		**43,220**				
			Ga	0.8000	0.8000	0.8084
			Mi	0.7258	0.7258	0.7341

^a^CCC: concordance correlation coefficient.

^b^ICC: intraclass coefficient correlation.

^c^PPMCC: Pearson product moment correlation coefficient.

^d^GA: Garmin Vivosmart HR^+^.

^e^Mi: Xiaomi Mi Band 2.

**Figure 2:**
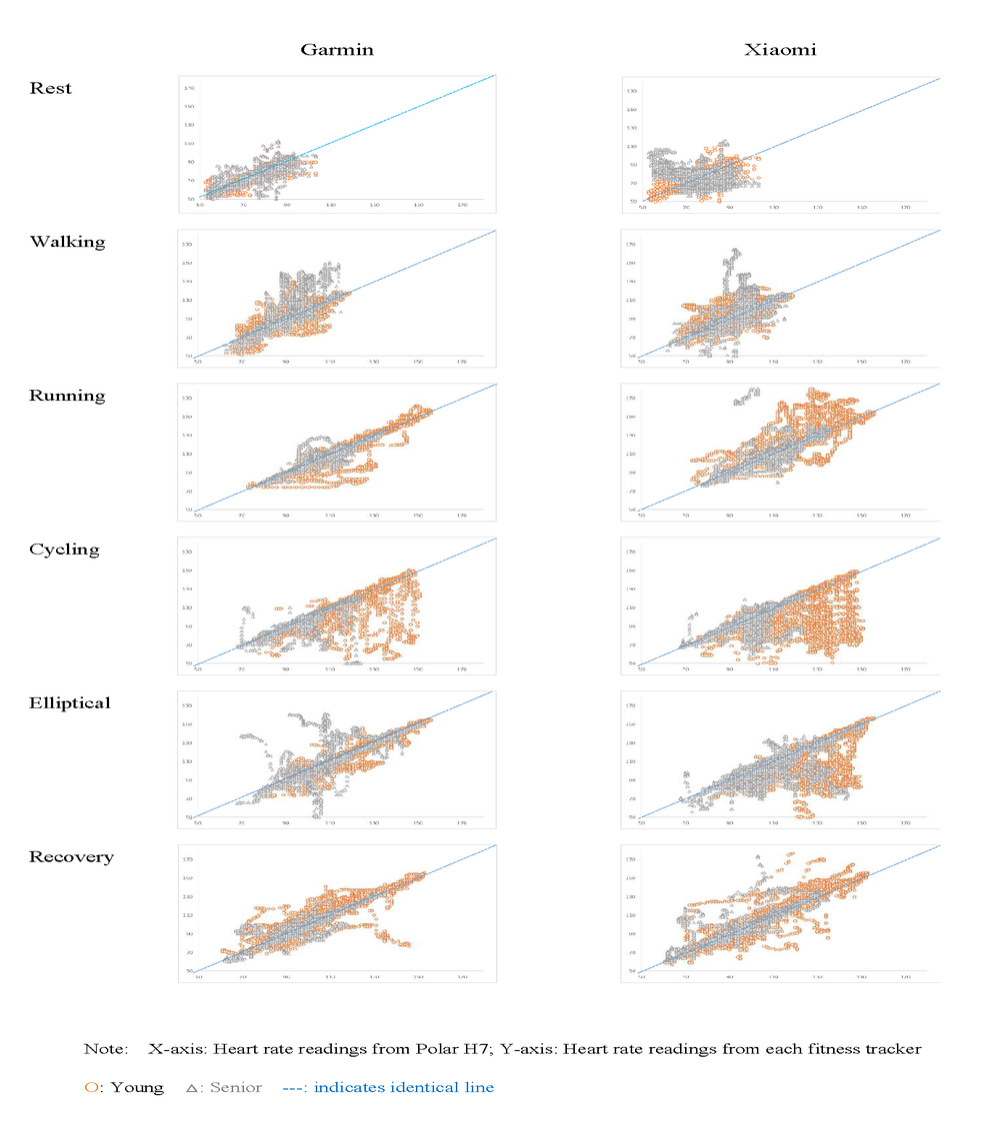
Scatter diagrams of the different phases of activities for different devices and groups.

To observe the overall trends and identify any apparent discrepancies in the correlation in different situations, each phase within the exercise sequence was plotted separately and color coded. The overlaid datasets of the different groups are represented in the scatter gram in Figure 2. Notably, the correlation of certain activities, such as cycling, was found to deviate from the criterion measurements much more frequently than activities such as walking. This was further confirmed using the Bland-Altman analysis (Table 1; see *Bland-Altman Analysis*).

### Bland-Altman Analysis

**Figure 3:**
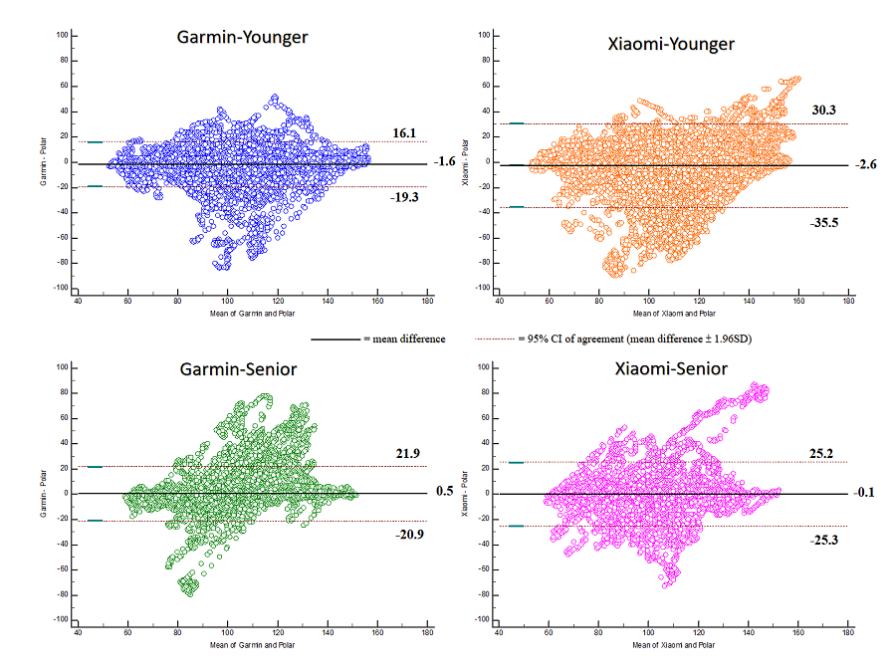
Bland-Altman plots of overall phases for different age groups and devices.

Bland-Altman plots indicating the mean difference in heart rate detection between Garmin or Xiaomi and Polar H7 criterion measure and levels of agreement with 95% CIs for the Young and Senior groups are illustrated in Figure 3. The complete Bland-Altman analysis dataset is presented in Table 1 (the Bland-Altman plot for each activity phase is provided in Multimedia Appendix 1). The data indicated that both test devices achieved relatively higher variations during cycling phases compared with other activities (Table 1). These results suggest that both devices tended to underreport heart rates in certain situations, consistent with previous observations [16,24]. Notably, the Xiaomi device significantly underestimated heart rates during cycling and elliptical phases in the Young group (−13.4 bpm and −13.3 bpm, respectively). Moreover, the differences between the upper and lower limits during the recovery phase (rest sitting between active phases) were greater than those during the resting phase (rest sitting in the beginning; Table 1). This implies that the variation of differences was greater at the transitional phases in which participants changed their activities from dynamic exercise to recovery, and thus, the degree of errors might decrease gradually if the participants stay in the rest position.

### Comparison of Correlation Tests

Various combinations of correlation tests are frequently adopted in evaluating the reliability or validity of examined devices [35]. As such, 3 independent statistical tests were employed in this study to compare whether the results from different correlation tests would deviate.

The obtained results (Table 2) revealed that the PPMCC test might compute a higher correlation coefficient than the CCC and ICC tests. The results of all the phases were quite identical; for example, the maximum difference was less than 0.01 (0.7258 and 0.7341 for Mi Band 2 in the Senior group). However, the difference between CCC or ICC and PPMCC was more obvious for activities; for example, a higher deviation was noted for activities such as cycling and elliptical exercise.

## Discussion

### Principal Findings

In line with previous studies [2,11,16,53], the combined results from this study indicated that both the Garmin and Xiaomi devices generally provided accurate heart rate readings. Both devices were also considered reliable in heart rate measurements with overall MAPE values below the 10% threshold. Notably, even though both devices achieved acceptable overall correlations in both age groups, they showed a tendency to modestly underestimate heart rates in many situations, as revealed by the Bland-Altman analysis. Similar findings were also reported in previous studies [11,12] and could represent a general characteristic of optical heart rate fitness trackers.

However, it is worth noting that significant discrepancies in device accuracy remained apparent between different physical activities. In general, these devices would be more accurate during sedentary behaviors such as sitting compared with active exercise [2]. Indeed, a previous study on a number of commercial wearable activity monitors have found that most devices exhibited low ICC values (r<0.5) when the activity intensity exceeded 100 watts in graded cycling exercise [13]. Similarly, our data revealed that the test devices generally had lower correlation coefficients and higher degrees of deviation during cycling and elliptical exercises compared with other activities.

In addition to activity intensity, several other studies have identified that motion artifacts during exercise were negatively correlated with the accuracy of PPG heart rate–monitoring systems [32,38,46,54-56]. For example, in an experiment conducted by Gillinov et al [2], the optical devices exhibited more accuracy for exercise with fewer arm motion artifacts (cycling and elliptical exercise with no arms movement). It is somewhat surprising that the data collected in this study indicated the opposite (as cycling produced less motion artifact than running). Nevertheless, Benedetto et al [12] found that the Fitbit charge 2 had poor ICC values (r=0.21) and underestimated the actual heart rate values when performing stationary cycling. Without further conclusions, users should be cautious when relying on optical heart rate readouts during various physical activities. Taken together, this study provides supporting evidence for a negative correlation between activity type and the accuracy of optical heart rate sensors but not between motion artifacts and the accuracy of optical heart rate sensors [2,13,24]. The precise mechanisms for such correlations currently remain unclear.

The profoundly expanding aging population worldwide is creating challenges for all sectors in the society. Promoting health condition of the older adult population and motivating them to engage in regular physical activity have become essential [57]. The adoption of new technology such as using health-related informatics technology (such as apps) or wearable fitness trackers is increasing [20,58,59], and the benefits are also observed in the senior population [20,60]. The fitness trackers validated in this study appear to exhibit similar accuracy for heart rate detection among different age groups.

**Figure 4:**
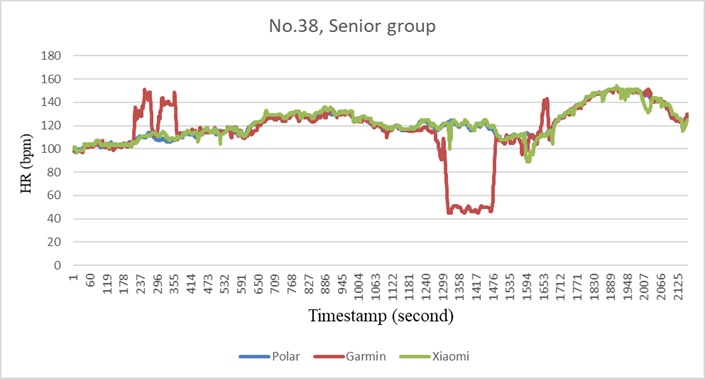
Subject-specific recording errors during the cycling phase.

Given its more thorough data acquisition method, this study had identified certain unexpected outliers. As shown in Figure 4, these extreme readings were unexpected, unpredictable, and transient. It is likely that these extreme readings did not represent the true heart rate values and that their displays were technical faults of the devices or the detection approach. Nonetheless, these random (or untrue) readings can skew the overall dataset and falsely represent the heart rate of an individual. Because these extreme heart rate readings were only observed for a short period, detecting these deviations while examining the heart rate readings every 15 seconds, every minute, or only at the end of the exercise, as in earlier studies, is difficult [11,15,16]. Given the transiency of such extreme readings, it is therefore recommended that future studies on optical heart rate sensors adopt a second-by-second approach demonstrated here and previously [12] to identify the outliers.

Previous studies have proposed the use of different statistical methods to analyze the data correlation. These include the MAPE test, the Bland-Altman analysis, the correlation PPMCC, ICC, and CCC tests [2,12,13,15,53]. To minimize the insufficiencies of individual statistical tests, this study examined the second-by-second heart rate readings using all of the mentioned correlation tests. Our results showed that when given the same dataset, PPMCC tests would typically derive higher values than ICC or CCC tests. Although all of the correlation coefficients have previously been adopted in other studies on optical devices, future research should exercise caution when selecting correlation tests and interpreting test results. That said, ICC and CCC should nonetheless be the preferred tests, as they were initially used to assess the interrater reliability in related validation studies [61,62]. Sartor et al [38] also supported the use of the CCC test for validating wrist-based heart rate monitors. Another study has proposed standardization of exercise protocols to ensure that the aggregate data were reproducible [51]. Thus, a standard set of examining methods and statistical analyses should be developed and adopted in future validation studies of optical heart rate sensors.

In conclusion, this study revealed that both the Garmin and Xiaomi optical heart rate sensors were capable of producing fairly accurate heart rate readings for both young and older adults. In particular, these devices achieved better accuracy during sedentary behaviors compared with physical activities. The heart rate reading accuracy of both devices was influenced by different types of physical activities. Consistently, the results echoed the previously reported tendency for heart rate underestimation during cycling and elliptical training in both of the devices. Notably, both devices exhibited the tendency to transiently display erroneous extreme readings. Thus, cautions should be exercised when using wrist-strapped fitness trackers to monitor the real-time heart rate during aerobic exercises.

### Limitations

This study was limited by several factors. First, the test devices were chosen because of their popularity in Asia and the availability of the broadcasting heart rate mode on these devices. However, different brands would usually be integrated with different PPG modules or algorithms, which could lead to discrepancies among the different optical heart rate devices [2,11]. This makes direct interpretations of findings on other optical heart rate devices using the current results more difficult. Although this study strived to retrieve the second-by-second data, the heart rate signals derived from various devices were complex, and the time lag problem existed between the investigational and reference devices [38]; in addition, owing to the trade secrets pertaining to the PPG signal-processing algorithms and the receiving apps, we could only assume that the second-by-second data are from the nearest previous beat-to-beat waveform signal to represent the heart rate readings. Nevertheless, the PPG sensor provided satisfactory readings when it was worn on the wrist than on other body parts. Second, the exercise intensity in this study was set at a submaximal level because of the various physical conditions of the participants. Thus, performance of these examining devices during more vigorous intensity exercises remains to be examined. In addition, this study only selected healthy participants, that is, participants without any cardiovascular diseases (eg, coronary artery disease or abnormal heart rhythms) or neurological disorders (eg, Parkinson disease or essential tremor) because the abnormal heart rate might interfere in the accuracy of comparison [63,64]. Hence, the results cannot be generalized to the overall older adult population. The validity of PPG fitness trackers for a population with major disorders, such as patients with cardiac disorders, requires further investigation.

### Suggestions

Future research on these topics should benefit from the standardization of the exercise protocol, selected statistical methods, and the threshold of acceptable accuracy. This will allow for better cross-study comparisons and more accurate interpretations [51]. Second, future studies can incorporate more participants with various health conditions to increase the representativeness of the cohort. Conducting multiple trials for the same cohort will control variability. This will also help identify erroneous readings, especially when they fall within the physiological range. For similar reasons, the second-by-second data acquisition method presented in this study should be adopted in all future studies. This will also help address the mechanisms of those conceivably erroneous displays. Third, future testing should include more contextual activities, such as outdoor walking, running, and cycling, to better mimic real-life events. This will allow for better comparisons of device performances under different settings.

### Conclusions

Overall, the results of this study indicate that both the Garmin and Xiaomi optical heart rate sensors exhibit acceptable heart rate–sensing accuracy for yellow skin tone population (Fitzpatrick skin type III or IV). Both devices perform similar to the Polar H7 chest-strapped heart rate monitor. The results also indicate that the sensing reliability of both the Garmin and Xiaomi devices can be influenced by different types of physical activities and that the Garmin device generally outperformed the Xiaomi device. The accuracy of both devices was not significantly affected by the age of users which implies that both devices are suitable for use in older adults. This has significant implications for the increasing aging population because PPG fitness trackers are inexpensive and use a noninvasive technology to provide information regarding various parameters and they have a great potential for telemedicine use considering remote or home health monitoring, assisting the older adult population to monitor their health [32].

The accuracy levels of both devices were negatively correlated with the level of activity intensity. For both devices, the measurement accuracy deteriorated in individuals while cycling. For unknown reasons, this study also reports the occurrence of extreme errors in these heart rate–sensing devices. These relevant findings imply that users or exercise practitioners should be cautious when using wrist-strapped fitness trackers to monitor exercise performance.

## Appendix

Multimedia Appendix 1 Bland-Altman plots of each phase for different groups and devices.

## Abbreviations

CCC: concordance correlation coefficient
ICC: intraclass coefficient correlation
MAE: mean absolute error
MAPE: mean absolute percentage error
PPG: photoplethysmography
PPMCC: Pearson product moment correlation coefficient

## References

[1] El-Amrawy F, Nounou MI (2015). Are currently available wearable devices for activity tracking and heart rate monitoring accurate, precise, and medically beneficial?. Healthc Inform Res.

[2] Gillinov S, Etiwy M, Wang R, Blackburn G, Phelan D, Gillinov AM, Houghtaling P, Javadikasgari H, Desai MY (2017). Variable accuracy of wearable heart rate monitors during aerobic exercise. Med Sci Sports Exerc.

[3] Thompson WR (2015). Worldwide survey of fitness trends for 2016. ACSMs Health Fit J.

[4] Thompson WR (2016). Worldwide survey of fitness trends for 2017. ACSMs Health Fit J.

[5] Thompson WR (2018). Worldwide survey of fitness trends for 2019. ACSMs Health Fit J.

[6] Thompson WR (2019). Worldwide survey of fitness trends for 2020. ACSMs Health Fit J.

[7] Laukkanen RM, Virtanen PK (1998). Heart rate monitors: state of the art. J Sports Sci.

[8] Böhm M, Reil J, Deedwania P, Kim JB, Borer JS (2015). Resting heart rate: risk indicator and emerging risk factor in cardiovascular disease. Am J Med.

[9] Challoner AV, Ramsay CA (1974). A photoelectric plethysmograph for the measurement of cutaneous blood flow. Phys Med Biol.

[10] Spierer DK, Rosen Z, Litman LL, Fujii K (2015). Validation of photoplethysmography as a method to detect heart rate during rest and exercise. J Med Eng Technol.

[11] Wallen MP, Gomersall SR, Keating SE, Wisløff U, Coombes JS (2016). Accuracy of heart rate watches: Implications for weight management. PLoS One.

[12] Benedetto S, Caldato C, Bazzan E, Greenwood DC, Pensabene V, Actis P (2018). Assessment of the Fitbit Charge 2 for monitoring heart rate. PLoS One.

[13] Boudreaux BD, Hebert EP, Hollander DB, Williams BM, Cormier CL, Naquin MR, Gillan WW, Gusew EE, Kraemer RR (2018). Validity of wearable activity monitors during cycling and resistance exercise. Med Sci Sports Exerc.

[14] Dooley EE, Golaszewski NM, Bartholomew JB (2017). Estimating accuracy at exercise intensities: A comparative study of self-monitoring heart rate and physical activity wearable devices. JMIR Mhealth Uhealth.

[15] Stahl SE, An H, Dinkel DM, Noble JM, Lee J (2016). How accurate are the wrist-based heart rate monitors during walking and running activities? Are they accurate enough?. BMJ Open Sport Exerc Med.

[16] Wang R, Blackburn G, Desai M, Phelan D, Gillinov L, Houghtaling P, Gillinov M (2017). Accuracy of wrist-worn heart rate monitors. JAMA Cardiol.

[17] Shih PC, Han K, Poole ES, Rosson MB, Carroll JM (2015). Use and Adoption Challenges of Wearable Activity Trackers. iConference 2015 Proceedings.

[18] Michaelis JR, Rupp MA, Kozachuk J, Ho B, Zapata-Ocampo D, McConnell DS, Smither JA (2016). Describing the user experience of wearable fitness technology through online product reviews. Proc Hum Factors Ergon Soc Annu Meet.

[19] Yang R, Shin E, Newman MW, Ackerman MS (2015). When Fitness Trackers Don't 'Fit': End-user Difficulties in the Assessment of Personal Tracking Device Accuracy. Proceedings of the 2015 ACM International Joint Conference on Pervasive and Ubiquitous Computing.

[20] Mercer K, Giangregorio L, Schneider E, Chilana P, Li M, Grindrod K (2016). Acceptance of commercially available wearable activity trackers among adults aged over 50 and with chronic illness: a mixed-methods evaluation. JMIR Mhealth Uhealth.

[21] Takacs J, Pollock C, Guenther J, Bahar M, Napier C, Hunt M (2014). Validation of the Fitbit One activity monitor device during treadmill walking. J Sci Med Sport.

[22] Kooiman TJ, Dontje ML, Sprenger SR, Krijnen WP, van der Schans CP, de Groot M (2015). Reliability and validity of ten consumer activity trackers. BMC Sports Sci Med Rehabil.

[23] Evenson KR, Goto MM, Furberg RD (2015). Systematic review of the validity and reliability of consumer-wearable activity trackers. Int J Behav Nutr Phys Act.

[24] Dondzila C, Lewis C, Lopez J, Parker T (2018). Congruent accuracy of wrist-worn activity trackers during controlled and free-living conditions. Int J Exerc Sci.

[25] Nelson MB, Kaminsky LA, Dickin DC, Montoye AH (2016). Validity of consumer-based physical activity monitors for specific activity types. Med Sci Sports Exerc.

[26] Wahl Y, Düking P, Droszez A, Wahl P, Mester J (2017). Criterion-validity of commercially available physical activity tracker to estimate step count, covered distance and energy expenditure during sports conditions. Front Physiol.

[27] Fortune E, Lugade V, Morrow M, Kaufman K (2014). Validity of using tri-axial accelerometers to measure human movement - Part II: Step counts at a wide range of gait velocities. Med Eng Phys.

[28] Lauritzen J, Muñoz A, Sevillano JL, Civit A (2013). The usefulness of activity trackers in elderly with reduced mobility: a case study. Stud Health Technol Inform.

[29] Parak J, Korhonen I (2014). Evaluation of Wearable Consumer Heart Rate Monitors Based on Photopletysmography. Proceedings of the 2014 36th Annual International Conference of the IEEE Engineering in Medicine and Biology Society.

[30] Preejith S, Alex A, Joseph J, Sivaprakasam M (2016). Design, Development and Clinical Validation of a Wrist-based Optical Heart Rate Monitor. Proceedings of the 2016 IEEE International Symposium on Medical Measurements and Applications (MeMeA).

[31] Fokkema T, Kooiman TJ, Krijnen WP, van der Schans CP, de Groot M (2017). Reliability and validity of ten consumer activity trackers depend on walking speed. Med Sci Sports Exerc.

[32] Allen J (2007). Photoplethysmography and its application in clinical physiological measurement. Physiol Meas.

[33] Gil E, Orini M, Bailón R, Vergara JM, Mainardi L, Laguna P (2010). Photoplethysmography pulse rate variability as a surrogate measurement of heart rate variability during non-stationary conditions. Physiol Meas.

[34] Schäfer A, Vagedes J (2013). How accurate is pulse rate variability as an estimate of heart rate variability? A review on studies comparing photoplethysmographic technology with an electrocardiogram. Int J Cardiol.

[35] Wright SP, Brown TS, Collier SR, Sandberg K (2017). How consumer physical activity monitors could transform human physiology research. Am J Physiol Regul Integr Comp Physiol.

[36] (2018). Leagle.

[37] (2016). Leagle.

[38] Sartor F, Papini G, Cox LG, Cleland J (2018). Methodological shortcomings of wrist-worn heart rate monitors validations. J Med Internet Res.

[39] Delgado R, Parák J, Tarniceriu A, Renevey P, Bertschi M, Korhonen I (2015). Evaluation of Accuracy and Reliability of PulseOn Optical Heart Rate Monitoring Device. Proceedings of 2015 37th Annual International Conference of the IEEE Engineering in Medicine and Biology Society (EMBC).

[40] McCullough JL, Kelly KM (2006). Prevention and treatment of skin aging. Ann N Y Acad Sci.

[41] Grimes PE, Sherrod Q, Grimes PE (2008). Structural and physiologic differences in the skin of darker racial ethnic groups. Aesthetics and Cosmetic Surgery for Darker Skin Types.

[42] Australian Radiation Protection and Nuclear Safety Agency.

[43] Zou GY (2012). Sample size formulas for estimating intraclass correlation coefficients with precision and assurance. Stat Med.

[44] Cheatham SW, Kolber MJ, Ernst MP (2015). Concurrent validity of resting pulse-rate measurements: a comparison of 2 smartphone applications, the polar H7 belt monitor, and a pulse oximeter with bluetooth. J Sport Rehabil.

[45] Gorny AW, Liew SJ, Tan CS, Müller-Riemenschneider F (2017). Fitbit Charge HR wireless heart rate monitor: validation study conducted under free-living conditions. JMIR Mhealth Uhealth.

[46] Lang M (2017). Beyond Fitbit: a critical appraisal of optical heart rate monitoring wearables and apps, their current limitations and legal implications. Alb LJ Sci Tech.

[47] Dorgo S, Robinson KM, Bader J (2009). The effectiveness of a peer-mentored older adult fitness program on perceived physical, mental, and social function. J Am Acad Nurse Pract.

[48] Prosser LA, Stanley CJ, Norman TL, Park HS, Damiano DL (2011). Comparison of elliptical training, stationary cycling, treadmill walking and overground walking. Electromyographic patterns. Gait Posture.

[49] Stanish HI, Temple VA (2012). Efficacy of a peer-guided exercise programme for adolescents with intellectual disability. J Appl Res Intellect Disabil.

[50] American College of Sports Medicine (2014). ACSM's Guidelines for Exercise Testing and Prescription. Ninth Edition.

[51] Bunn JA, Navalta JW, Fountaine CJ, Reece JD (2018). Current state of commercial wearable technology in Physical Activity Monitoring 2015-2017. Int J Exerc Sci.

[52] Koo TK, Li MY (2016). A guideline of selecting and reporting intraclass correlation coefficients for reliability research. J Chiropr Med.

[53] Sartor F, Gelissen J, van Dinther R, Roovers D, Papini GB, Coppola G (2018). Wrist-worn optical and chest strap heart rate comparison in a heterogeneous sample of healthy individuals and in coronary artery disease patients. BMC Sports Sci Med Rehabil.

[54] Alqaraawi A, Alwosheel A, Alasaad A (2016). Heart rate variability estimation in photoplethysmography signals using Bayesian learning approach. Healthc Technol Lett.

[55] Sweeney KT, Ward TE, McLoone SF (2012). Artifact removal in physiological signals--practices and possibilities. IEEE Trans Inf Technol Biomed.

[56] Ahmadi AK, Moradi P, Malihi M, Karimi S, Shamsollahi MB (2015). Heart Rate Monitoring During Physical Exercise Using Wrist-Type Photoplethysmographic (PPG) Signals. Proceedings of 2015 37th Annual International Conference of the IEEE Engineering in Medicine and Biology Society.

[57] Nied R, Franklin B (2002). Promoting and prescribing exercise for the elderly. Am Fam Physician.

[58] McMahon SK, Lewis B, Oakes M, Guan W, Wyman JF, Rothman AJ (2016). Older adults' experiences using a commercially available monitor to self-track their physical activity. JMIR Mhealth Uhealth.

[59] Heart T, Kalderon E (2013). Older adults: are they ready to adopt health-related ICT?. Int J Med Inform.

[60] Rasche P, Wille M, Theis S, Schäefer K, Schlick C, Mertens A (2015). Activity Tracker and Elderly. Proceedings of the 2015 IEEE International Conference on Computer and Information Technology; Ubiquitous Computing and Communications; Dependable, Autonomic and Secure Computing; Pervasive Intelligence and Computing.

[61] Chen CC, Barnhart HX (2008). Comparison of ICC and CCC for assessing agreement for data without and with replications. Comput Stat Data Anal.

[62] Chen CC, Barnhart HX (2013). Assessing agreement with intraclass correlation coefficient and concordance correlation coefficient for data with repeated measures. Comput Stat Data Anal.

[63] Finsterer J, Wahbi K (2014). CNS-disease affecting the heart: brain-heart disorders. J Neurol Sci.

[64] Dyer AR, Persky V, Stamler J, Paul O, Shekelle RB, Berkson DM, Lepper M, Schoenberger JA, Lindberg HA (1980). Heart rate as a prognostic factor for coronary heart disease and mortality: findings in three Chicago epidemiologic studies. Am J Epidemiol.

