# Supplementary material for: Accuracy of Optical Heart Rate Sensing Technology in Wearable Fitness Trackers for Young and Older Adults: Validation and Comparison Study
Source: JMIR Mhealth Uhealth. 2020 Apr 28;8(4):e14707. doi: 10.2196/14707 (PMC7218601; doi:10.2196/14707)
Supplement: Multimedia Appendix 1 [file mhealth_v8i4e14707_app1.docx]

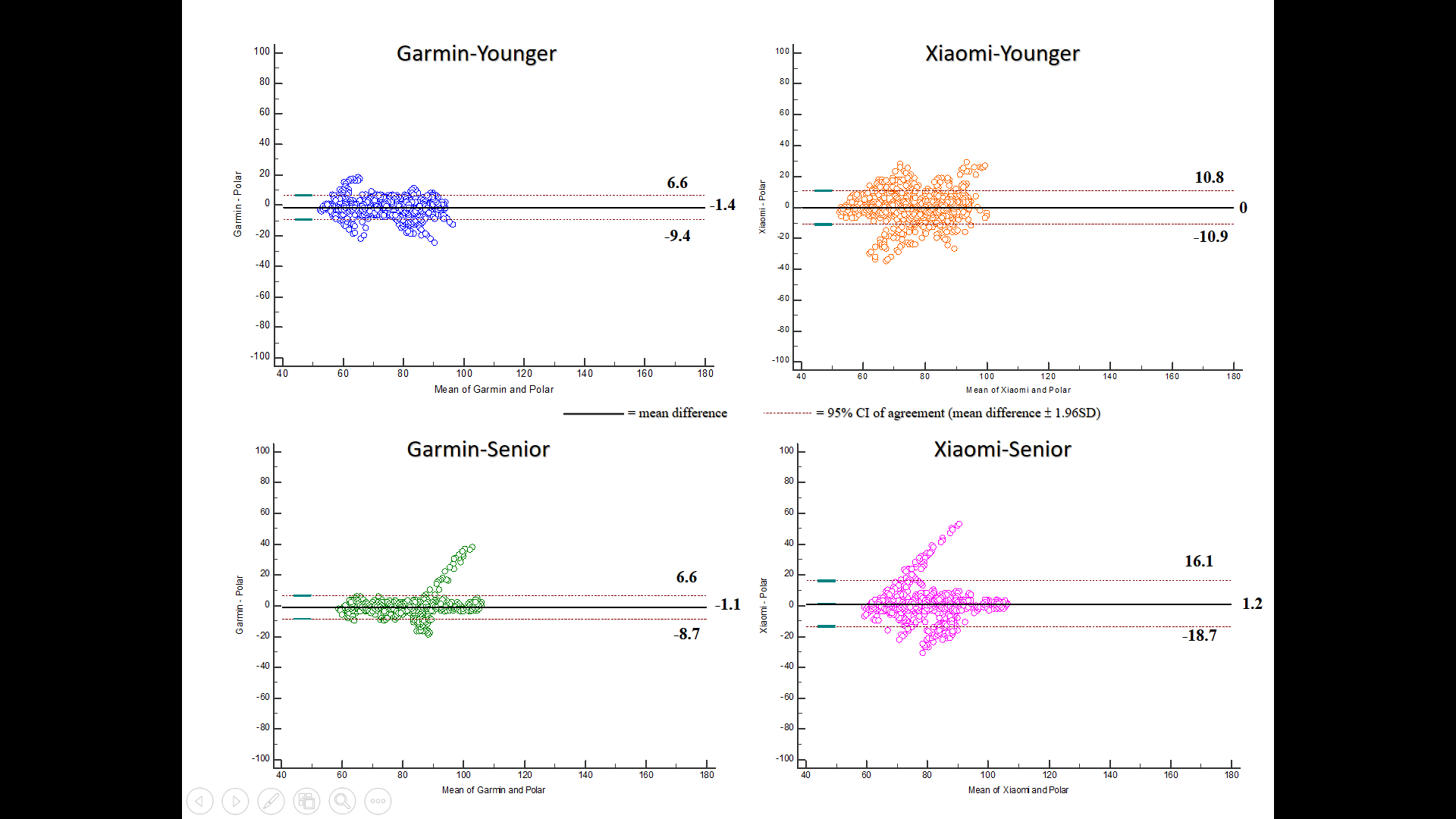


Supplement Figure 1

Bland-Altman Plots of rest phase for different groups and devices


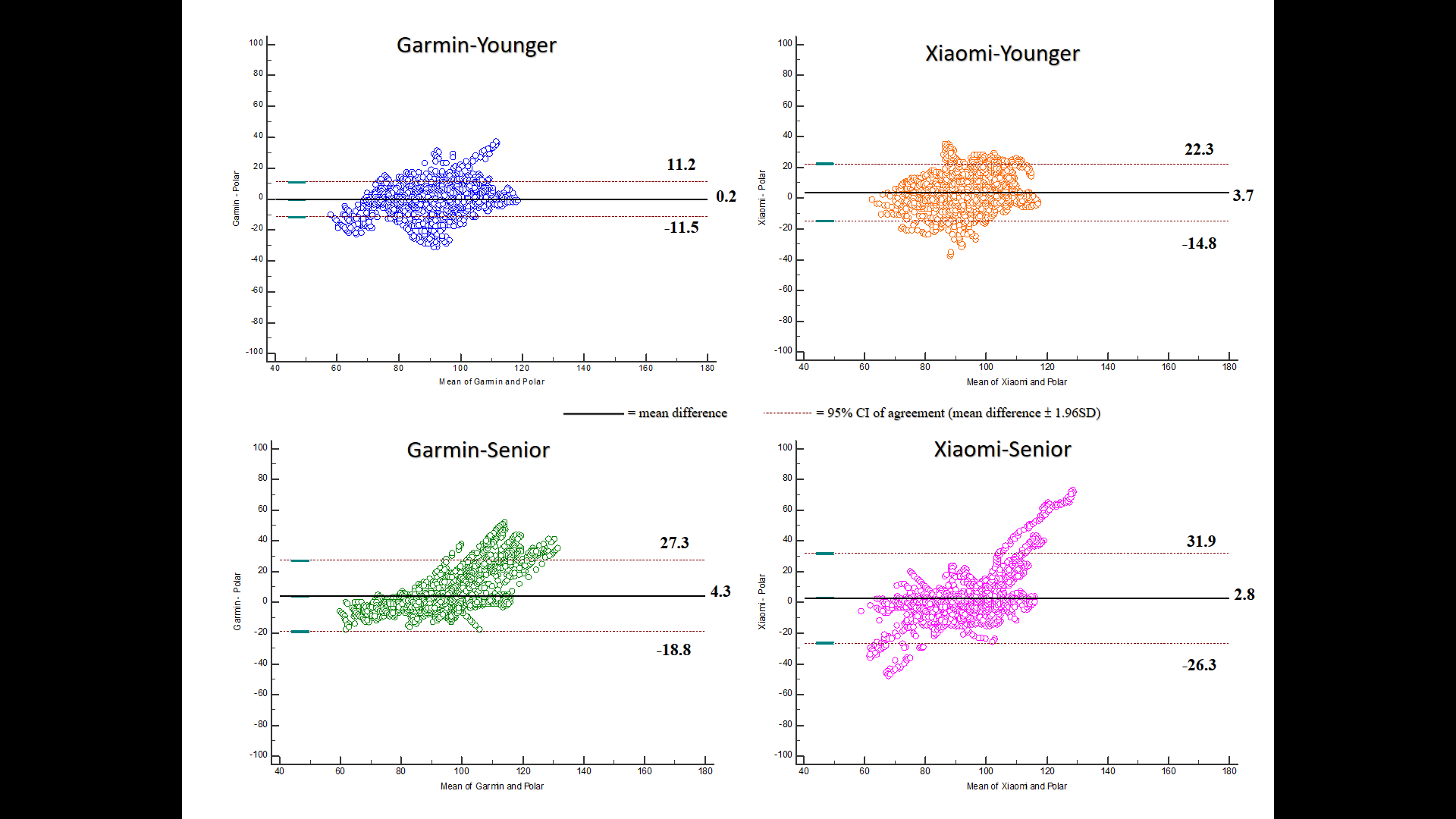


Supplement Figure 2

Bland-Altman Plots of walking phase for different groups and devices


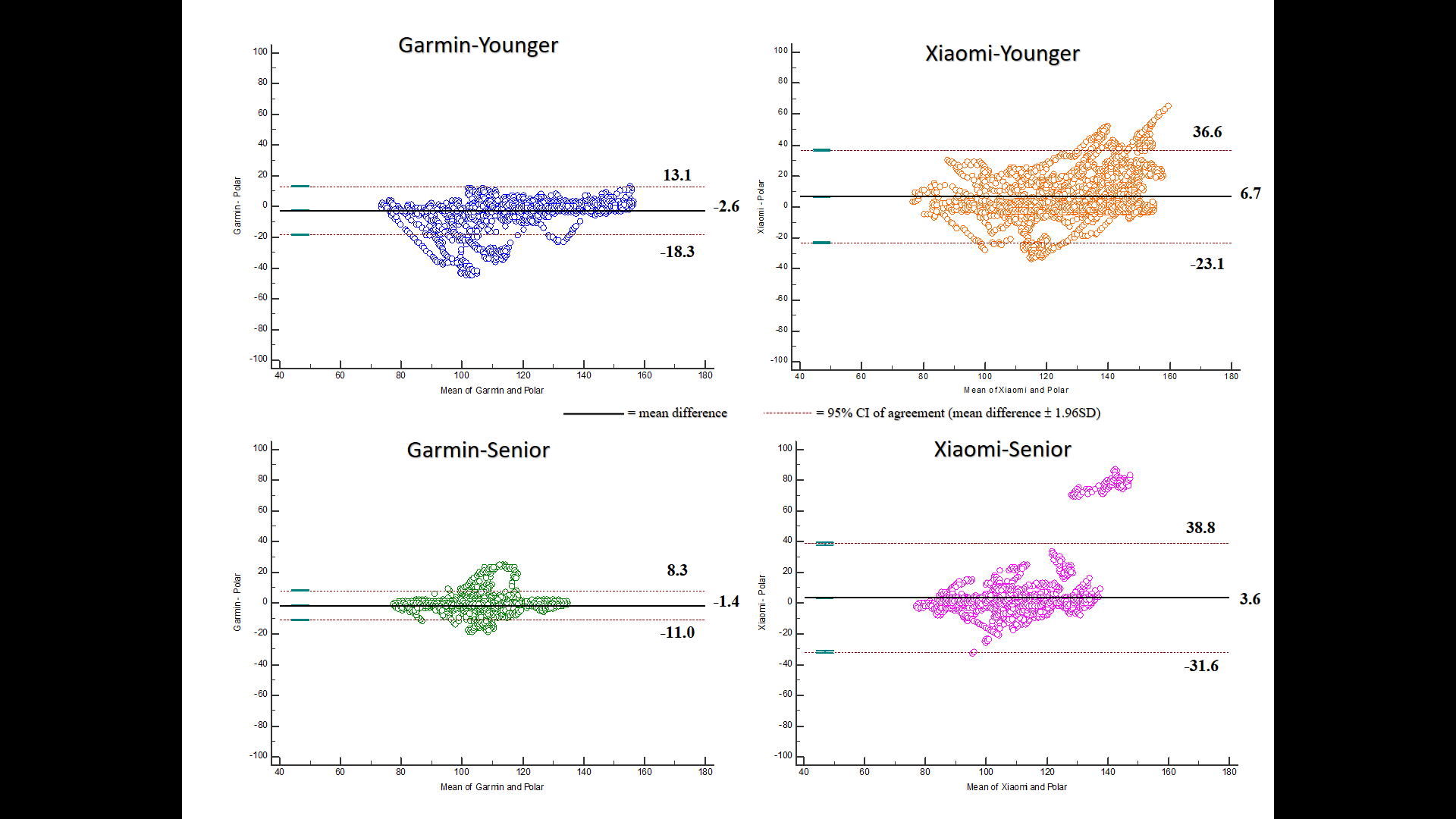


Supplement Figure 3

Bland-Altman Plots of running phase for different groups and devices


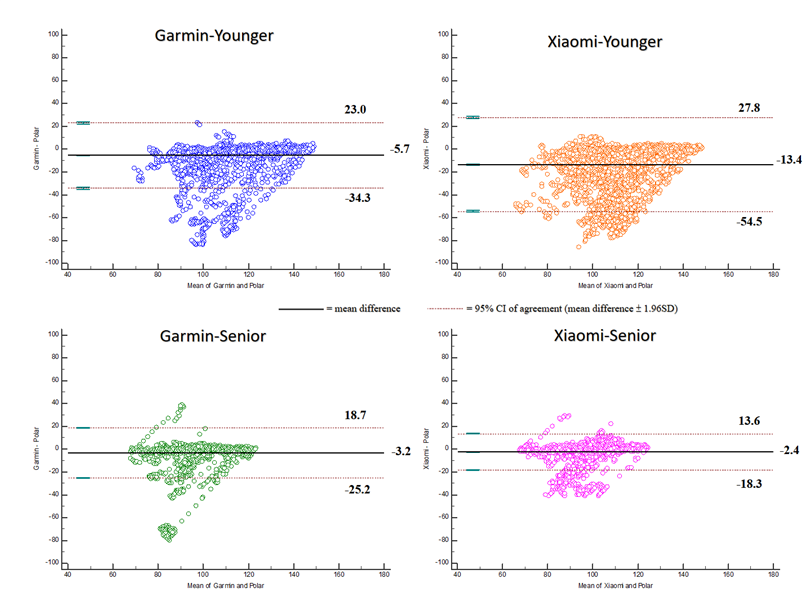


Supplement Figure 4

Bland-Altman Plots of cycling phase for different groups and devices


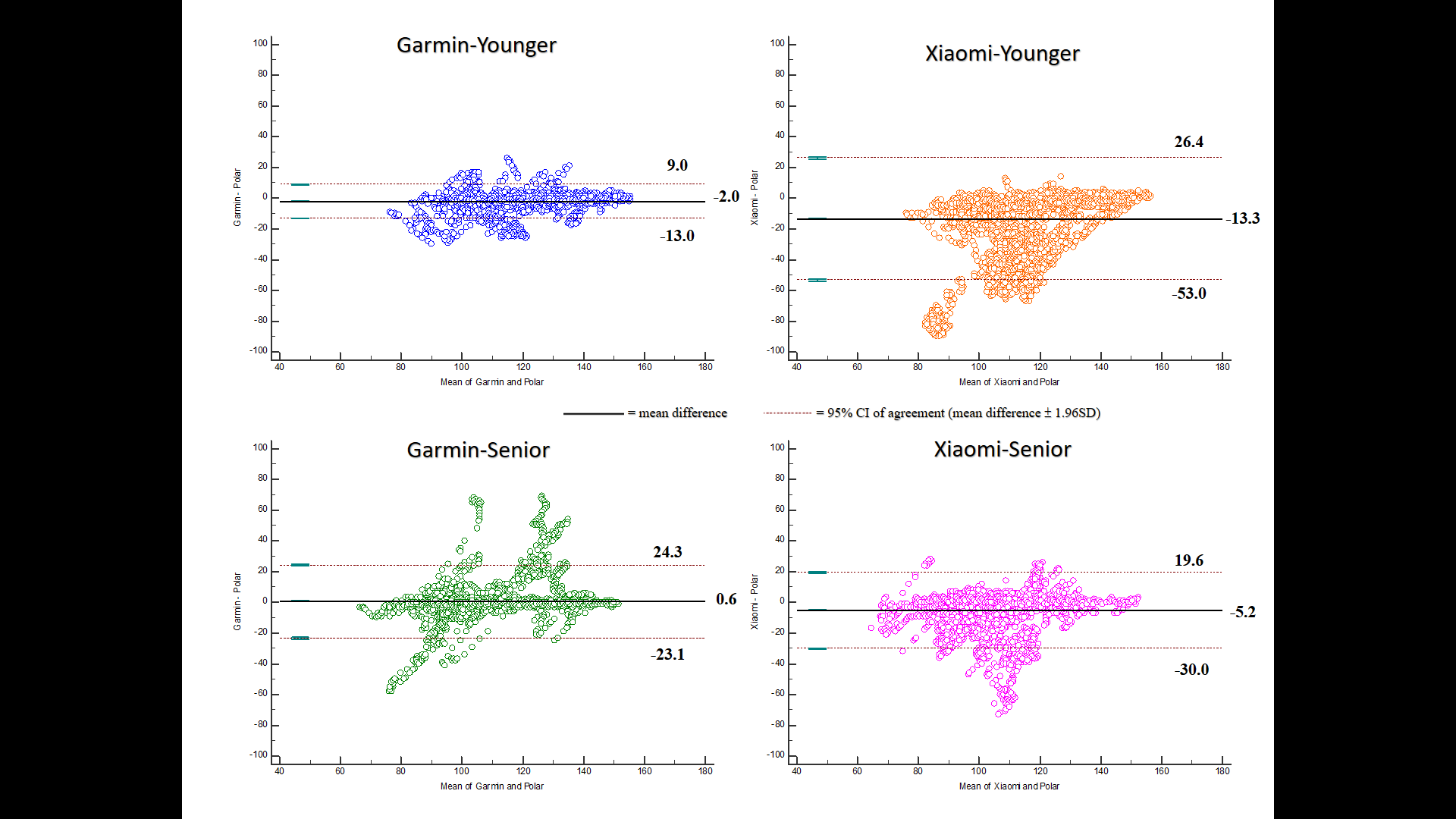


Supplement Figure 5

Bland-Altman Plots of elliptical exercise phase for different groups and devices


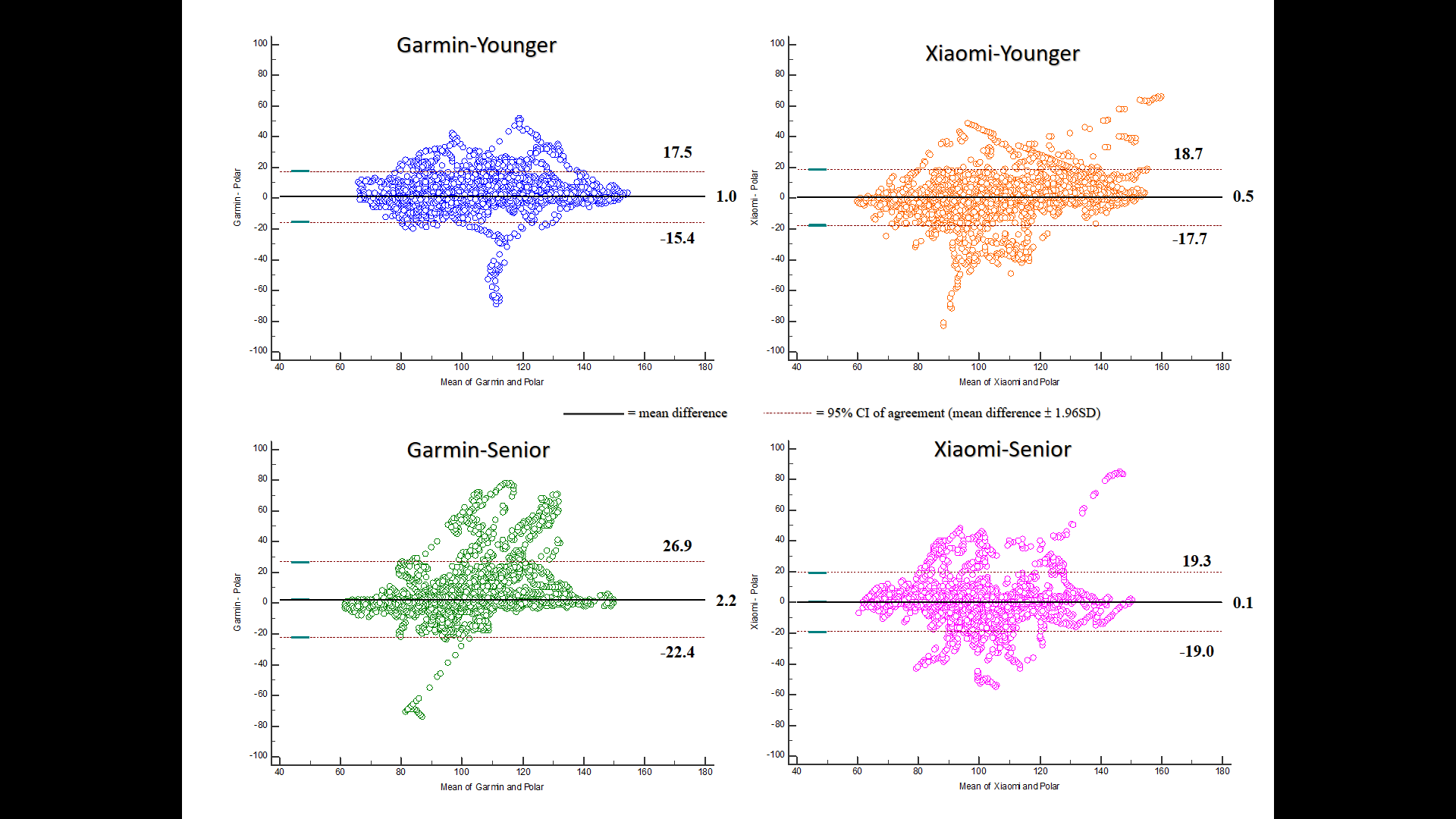


Supplement Figure 6

Bland-Altman Plots of recovery phase for different groups and devices
